# 5-(4-Chloro­phen­yl)-1-methyl-3-oxocyclo­hexa­necarbonitrile

**DOI:** 10.1107/S1600536808012816

**Published:** 2008-05-07

**Authors:** R. T. Sabapathy Mohan, S. Kamatchi, M. Subramanyam, A. Thiruvalluvar, A. Linden

**Affiliations:** a Department of Chemistry, Annamalai University, Annamalai Nagar 608 002, Tamil Nadu, India; b PG Research Department of Physics, Rajah Serfoji Government College (Autonomous), Thanjavur 613 005, Tamil Nadu, India; c Institute of Organic Chemistry, University of Zürich, Winterthurerstrasse 190, CH-8057 Zürich, Switzerland

## Abstract

In the title mol­ecule, C_14_H_14_ClNO, the cyclo­hexane ring adopts a chair conformation. The cyano group and the methyl group have axial and equatorial orientations, respectively. The benzene ring has an equatorial orientation. A C—H⋯π inter­action involving the benzene ring is found in the crystal structure.

## Related literature

Subramanyam *et al.* (2007*a* ▶,*b* ▶) and Thiruvalluvar *et al.* (2007 ▶) have reported the crystal structures of substituted cyclo­hexane derivatives, in which the cyclo­hexane rings are in a chair conformation.
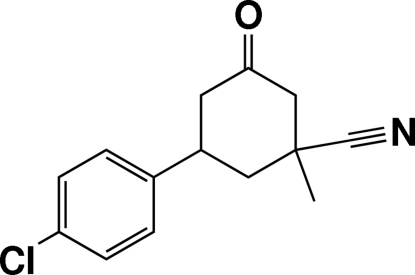


## Experimental

### 

#### Crystal data

C_14_H_14_ClNO
*M* *_r_* = 247.71
Monoclinic, 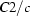

*a* = 23.3358 (6) Å
*b* = 6.0031 (2) Å
*c* = 20.8948 (6) Å
β = 122.386 (2)°
*V* = 2471.81 (14) Å^3^
*Z* = 8
Mo *K*α radiation
μ = 0.29 mm^−1^
*T* = 160 (1) K
0.28 × 0.20 × 0.18 mm

#### Data collection

Nonius KappaCCD area-detector diffractometer
Absorption correction: multi-scan (Blessing, 1995 ▶) *T*_min_ = 0.877, *T*_max_ = 0.956
28232 measured reflections
2822 independent reflections
2211 reflections with *I* > 2σ(*I*)
*R*_int_ = 0.058

#### Refinement

*R*[*F* ^2^ > 2σ(*F* ^2^)] = 0.040
*wR*(*F* ^2^) = 0.107
*S* = 1.05
2822 reflections
154 parameters
H-atom parameters constrained
Δρ_max_ = 0.26 e Å^−3^
Δρ_min_ = −0.32 e Å^−3^

### 

Data collection: *COLLECT* (Nonius, 2000 ▶); cell refinement: *DENZO-SMN* (Otwinowski & Minor, 1997 ▶); data reduction: *DENZO-SMN* and *SCALEPACK* (Otwinowski & Minor, 1997 ▶); program(s) used to solve structure: *SHELXS97* (Sheldrick, 2008 ▶); program(s) used to refine structure: *SHELXL97* (Sheldrick, 2008 ▶); molecular graphics: *ORTEP-3* (Farrugia, 1997 ▶); software used to prepare material for publication: *PLATON* (Spek, 2003 ▶).

## Supplementary Material

Crystal structure: contains datablocks global, I. DOI: 10.1107/S1600536808012816/ww2118sup1.cif

Structure factors: contains datablocks I. DOI: 10.1107/S1600536808012816/ww2118Isup2.hkl

Additional supplementary materials:  crystallographic information; 3D view; checkCIF report

## Figures and Tables

**Table 1:** Hydrogen-bond geometry (Å, °)

*D*—H⋯*A*	*D*—H	H⋯*A*	*D*⋯*A*	*D*—H⋯*A*
C4—H4*B*⋯*Cg*^i^	0.99	2.60	3.5333 (18)	157

Symmetry code: (i) 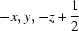. *Cg* is the centroid of the benzene ring.

**Fig. 1.**
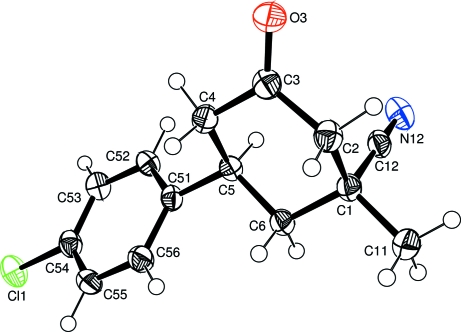
The molecular structure of the title compound, showing the atom-numbering scheme and displacement ellipsoids drawn at the 50% probability level. Hydrogen atoms are represented by spheres of arbitrary radius.

## supplementary crystallographic information

### Comment

Subramanyam *et al.* (2007*a*,b) and Thiruvalluvar *et al.* (2007) have reported the crystal structures of substituted cyclohexane derivatives, in which the cyclohexane rings are in chair conformation. The molecular structure of the title compound, with atomic numbering scheme, is shown in Fig. 1. The cyclohexane ring adopts a chair conformation. The cyano group and the methyl group at position 1 have axial and equatorial orientations respectively. The benzene ring at position 5 has an equatorial orientation. A C4—H4B···π(-*x*, *y*, 1/2 - *z*) interaction involving the benzene ring is found in the structure. No classical hydrogen bonds are found in the crystal structure.

### Experimental

A mixture of 5–4'-chlorophenyl-3-methylcyclohex-2-enone (6.40 g, 0.02 mol), potassium cyanide (2.60 g, 0.04 mol), ammonium chloride (1.59 g, 0.03 mol), dimethyl formamide (50 ml) and water (2 ml) was heated with stirring for 16–18 h at 353 K. The reaction mixture was cooled to room temperature and poured into water. The product was extracted with CH_2_Cl_2_ (3x10 ml) and the organic layer was dried, evaporated and purified by column chromatography (hexane-EtOAc, 4.5:1 *v*/*v*). The yield of the isolated product was 4.30 g (87%).

### Refinement

H atoms were positioned geometrically and allowed to ride on their parent atoms, with C—H = 0.95 Å for C*sp*^2^, 0.98 Å for methyl C, 0.99 Å for methylene C and 1.00 Å for methine C; *U*_iso_(H) = *xU*_eq_(carrier atom), where *x* = 1.5 for methyl and 1.2 for all other C atoms

### Crystal data

**Table:**

C_14_H_14_ClNO	*F*_000_ = 1040
*M**_r_* = 247.71	*D*_x_ = 1.331 Mg m^−^^3^
Monoclinic, *C*2/*c*	Melting point: 358 K
Hall symbol: -C 2yc	Mo *K*α radiation λ = 0.71073 Å
*a* = 23.3358 (6) Å	Cell parameters from 31960 reflections
*b* = 6.0031 (2) Å	θ = 2.0–27.5º
*c* = 20.8948 (6) Å	µ = 0.29 mm^−^^1^
β = 122.386 (2)º	*T* = 160 (1) K
*V* = 2471.81 (14) Å^3^	Prism, colourless
*Z* = 8	0.28 × 0.20 × 0.18 mm

### Data collection

**Table:**

Nonius KappaCCD area-detector diffractometer	2822 independent reflections
Radiation source: Nonius FR590 sealed tube generator	2211 reflections with *I* > 2σ(*I*)
Monochromator: horizontally mounted graphite crystal	*R*_int_ = 0.058
Detector resolution: 9 pixels mm^-1^	θ_max_ = 27.5º
*T* = 160(1) K	θ_min_ = 2.1º
φ and ω scans with κ offsets	*h* = −30→29
Absorption correction: multi-scan(Blessing, 1995)	*k* = −7→7
*T*_min_ = 0.877, *T*_max_ = 0.956	*l* = −27→27
28232 measured reflections	

### Refinement

**Table:**

Refinement on *F*^2^	Secondary atom site location: difference Fourier map
Least-squares matrix: full	Hydrogen site location: inferred from neighbouring sites
*R*[*F*^2^ > 2σ(*F*^2^)] = 0.040	H-atom parameters constrained
*wR*(*F*^2^) = 0.107	*w* = 1/[σ^2^(*F*_o_^2^) + (0.0525*P*)^2^ + 1.7764*P*] where *P* = (*F*_o_^2^ + 2*F*_c_^2^)/3
*S* = 1.05	(Δ/σ)_max_ < 0.001
2822 reflections	Δρ_max_ = 0.26 e Å^−^^3^
154 parameters	Δρ_min_ = −0.31 e Å^−^^3^
Primary atom site location: structure-invariant direct methods	Extinction correction: none

### Special details

**Table:**

Experimental. Cooling Device: Oxford Cryosystems Cryostream 700 Crystal mount: glued on a glass fibre Mosaicity (°.): 0.793 (2) Frames collected: 394 Seconds exposure per frame: 16 Degrees rotation per frame: 1.6 Crystal-Detector distance (mm): 30.0
Geometry. Bond distances, angles *etc*. have been calculated using the rounded fractional coordinates. All su's are estimated from the variances of the (full) variance-covariance matrix. The cell e.s.d.'s are taken into account in the estimation of distances, angles and torsion angles
Refinement. Refinement of *F*^2^ against ALL reflections. The weighted *R*-factor *wR* and goodness of fit *S* are based on *F*^2^, conventional *R*-factors *R* are based on *F*, with *F* set to zero for negative *F*^2^. The threshold expression of *F*^2^ > σ(*F*^2^) is used only for calculating *R*-factors(gt) *etc*. and is not relevant to the choice of reflections for refinement. *R*-factors based on *F*^2^ are statistically about twice as large as those based on *F*, and *R*- factors based on ALL data will be even larger.

### Fractional atomic coordinates and isotropic or equivalent isotropic displacement parameters (Å^2^)

**Table:**

	*x*	*y*	*z*	*U*_iso_*/*U*_eq_	
Cl1	−0.25175 (2)	0.41436 (8)	0.08818 (3)	0.0406 (2)	
O3	0.15529 (6)	1.0355 (2)	0.24871 (7)	0.0401 (4)	
N12	0.08284 (8)	0.8728 (3)	0.02081 (8)	0.0387 (5)	
C1	0.12125 (8)	0.5713 (3)	0.12750 (9)	0.0257 (5)	
C2	0.16797 (8)	0.6816 (3)	0.20566 (9)	0.0296 (5)	
C3	0.13140 (8)	0.8524 (3)	0.22416 (9)	0.0290 (5)	
C4	0.06351 (8)	0.7819 (3)	0.21034 (9)	0.0292 (5)	
C5	0.01764 (7)	0.6715 (3)	0.13228 (8)	0.0241 (4)	
C6	0.05647 (8)	0.4842 (3)	0.12188 (9)	0.0264 (5)	
C11	0.15935 (9)	0.3848 (3)	0.11531 (10)	0.0350 (5)	
C12	0.10064 (8)	0.7426 (3)	0.06780 (9)	0.0277 (5)	
C51	−0.04910 (7)	0.6001 (3)	0.12231 (8)	0.0239 (4)	
C52	−0.10261 (8)	0.7514 (3)	0.09191 (8)	0.0270 (5)	
C53	−0.16454 (8)	0.6975 (3)	0.08277 (9)	0.0293 (5)	
C54	−0.17302 (8)	0.4867 (3)	0.10337 (9)	0.0289 (5)	
C55	−0.12083 (9)	0.3334 (3)	0.13444 (10)	0.0322 (5)	
C56	−0.05897 (8)	0.3916 (3)	0.14389 (10)	0.0304 (5)	
H2A	0.18685	0.56475	0.24527	0.0355*	
H2B	0.20638	0.75446	0.20642	0.0355*	
H4A	0.04006	0.91413	0.21396	0.0350*	
H4B	0.07101	0.67576	0.25040	0.0350*	
H5	0.00699	0.78708	0.09293	0.0289*	
H6A	0.02664	0.41375	0.07173	0.0316*	
H6B	0.06906	0.36910	0.16120	0.0316*	
H11A	0.17294	0.27012	0.15427	0.0524*	
H11B	0.19981	0.44642	0.11878	0.0524*	
H11C	0.12964	0.31858	0.06508	0.0524*	
H52	−0.09668	0.89480	0.07705	0.0325*	
H53	−0.20035	0.80334	0.06276	0.0353*	
H55	−0.12702	0.19013	0.14917	0.0386*	
H56	−0.02282	0.28695	0.16550	0.0365*	

### Atomic displacement parameters (Å^2^)

**Table:**

	*U*^11^	*U*^22^	*U*^33^	*U*^12^	*U*^13^	*U*^23^
Cl1	0.0277 (2)	0.0501 (3)	0.0477 (3)	−0.0075 (2)	0.0227 (2)	0.0017 (2)
O3	0.0388 (7)	0.0398 (7)	0.0447 (7)	−0.0129 (6)	0.0243 (6)	−0.0142 (6)
N12	0.0368 (8)	0.0456 (9)	0.0359 (8)	−0.0065 (7)	0.0210 (7)	0.0043 (7)
C1	0.0237 (8)	0.0280 (8)	0.0280 (8)	−0.0011 (6)	0.0156 (7)	−0.0011 (6)
C2	0.0240 (8)	0.0357 (9)	0.0279 (8)	−0.0012 (7)	0.0131 (7)	−0.0017 (7)
C3	0.0287 (8)	0.0343 (9)	0.0220 (7)	−0.0040 (7)	0.0122 (7)	−0.0022 (7)
C4	0.0299 (8)	0.0316 (9)	0.0297 (8)	−0.0017 (7)	0.0184 (7)	−0.0036 (7)
C5	0.0242 (7)	0.0249 (8)	0.0253 (7)	−0.0005 (6)	0.0146 (6)	0.0019 (6)
C6	0.0257 (8)	0.0276 (8)	0.0287 (8)	−0.0037 (6)	0.0165 (7)	−0.0023 (7)
C11	0.0339 (9)	0.0342 (9)	0.0438 (10)	0.0003 (7)	0.0255 (8)	−0.0037 (8)
C12	0.0248 (8)	0.0338 (9)	0.0284 (8)	−0.0071 (7)	0.0169 (7)	−0.0056 (7)
C51	0.0239 (7)	0.0286 (8)	0.0215 (7)	−0.0019 (6)	0.0136 (6)	−0.0010 (6)
C52	0.0272 (8)	0.0285 (8)	0.0254 (8)	−0.0012 (7)	0.0141 (6)	0.0037 (6)
C53	0.0242 (8)	0.0352 (9)	0.0260 (8)	0.0019 (7)	0.0117 (7)	0.0038 (7)
C54	0.0240 (8)	0.0365 (9)	0.0276 (8)	−0.0071 (7)	0.0147 (7)	−0.0036 (7)
C55	0.0348 (9)	0.0265 (8)	0.0423 (10)	−0.0033 (7)	0.0254 (8)	0.0014 (7)
C56	0.0299 (8)	0.0273 (8)	0.0394 (9)	0.0032 (7)	0.0221 (8)	0.0047 (7)

### Geometric parameters (Å, °)

**Table:**

Cl1—C54	1.744 (2)	C54—C55	1.380 (3)
O3—C3	1.215 (2)	C55—C56	1.393 (3)
N12—C12	1.145 (2)	C2—H2A	0.9900
C1—C2	1.545 (2)	C2—H2B	0.9900
C1—C6	1.544 (3)	C4—H4A	0.9900
C1—C11	1.533 (3)	C4—H4B	0.9900
C1—C12	1.484 (2)	C5—H5	1.0000
C2—C3	1.511 (3)	C6—H6A	0.9900
C3—C4	1.511 (3)	C6—H6B	0.9900
C4—C5	1.541 (2)	C11—H11A	0.9800
C5—C6	1.531 (3)	C11—H11B	0.9800
C5—C51	1.518 (3)	C11—H11C	0.9800
C51—C52	1.392 (3)	C52—H52	0.9500
C51—C56	1.391 (3)	C53—H53	0.9500
C52—C53	1.391 (3)	C55—H55	0.9500
C53—C54	1.385 (3)	C56—H56	0.9500
			
Cl1···N12^i^	3.335 (2)	H4B···C51^ii^	3.0000
Cl1···C12^i^	3.397 (2)	H4B···C52^ii^	3.0000
Cl1···H2A^ii^	3.0900	H4B···C53^ii^	2.9600
O3···H6B^iii^	2.7300	H4B···C54^ii^	2.9200
O3···H11A^iii^	2.6300	H4B···C55^ii^	2.8900
O3···H55^iv^	2.7100	H4B···C56^ii^	2.9200
N12···Cl1^v^	3.335 (2)	H5···N12	2.9200
N12···H5	2.9200	H5···C12	2.5200
N12···H6A^vi^	2.8200	H5···H52	2.3500
N12···H52^vii^	2.6300	H6A···C56	3.0800
N12···H11C^iii^	2.8500	H6A···H11C	2.5500
C4···C12	3.527 (3)	H6A···N12^vi^	2.8200
C12···C4	3.527 (3)	H6A···C12^vi^	2.9900
C12···Cl1^v^	3.397 (2)	H6B···O3^ix^	2.7300
C6···H56	2.7300	H6B···C56	2.8100
C12···H5	2.5200	H6B···H11A	2.5700
C12···H6A^vi^	2.9900	H6B···H56	2.2500
C51···H4B^ii^	3.0000	H11A···O3^ix^	2.6300
C52···H4A	3.0700	H11A···H2A	2.4900
C52···H55^iii^	3.0700	H11A···H6B	2.5700
C52···H4B^ii^	3.0000	H11B···H2B	2.5500
C52···H11C^vi^	3.0200	H11C···N12^ix^	2.8500
C53···H4B^ii^	2.9600	H11C···H6A	2.5500
C53···H53^viii^	2.9900	H11C···C52^vi^	3.0200
C54···H4B^ii^	2.9200	H52···C55^iii^	3.0700
C55···H52^ix^	3.0700	H52···H5	2.3500
C55···H4B^ii^	2.8900	H52···N12^vii^	2.6300
C56···H6A	3.0800	H53···C53^viii^	2.9900
C56···H6B	2.8100	H53···H53^viii^	2.4800
C56···H4B^ii^	2.9200	H55···C52^ix^	3.0700
H2A···H11A	2.4900	H55···O3^x^	2.7100
H2A···Cl1^ii^	3.0900	H56···C6	2.7300
H2B···H11B	2.5500	H56···H4A^ix^	2.5700
H4A···C52	3.0700	H56···H6B	2.2500
H4A···H56^iii^	2.5700		
			
C2—C1—C6	109.48 (15)	C3—C2—H2B	109.00
C2—C1—C11	110.17 (15)	H2A—C2—H2B	108.00
C2—C1—C12	108.61 (15)	C3—C4—H4A	109.00
C6—C1—C11	111.80 (15)	C3—C4—H4B	109.00
C6—C1—C12	107.91 (15)	C5—C4—H4A	109.00
C11—C1—C12	108.79 (15)	C5—C4—H4B	109.00
C1—C2—C3	112.70 (16)	H4A—C4—H4B	108.00
O3—C3—C2	121.85 (19)	C4—C5—H5	107.00
O3—C3—C4	122.09 (18)	C6—C5—H5	107.00
C2—C3—C4	116.05 (15)	C51—C5—H5	107.00
C3—C4—C5	112.68 (15)	C1—C6—H6A	109.00
C4—C5—C6	109.74 (14)	C1—C6—H6B	109.00
C4—C5—C51	110.28 (14)	C5—C6—H6A	109.00
C6—C5—C51	114.51 (15)	C5—C6—H6B	109.00
C1—C6—C5	111.74 (15)	H6A—C6—H6B	108.00
N12—C12—C1	178.0 (2)	C1—C11—H11A	109.00
C5—C51—C52	118.81 (16)	C1—C11—H11B	109.00
C5—C51—C56	123.05 (16)	C1—C11—H11C	109.00
C52—C51—C56	118.12 (18)	H11A—C11—H11B	109.00
C51—C52—C53	121.54 (17)	H11A—C11—H11C	109.00
C52—C53—C54	118.75 (18)	H11B—C11—H11C	109.00
Cl1—C54—C53	118.89 (15)	C51—C52—H52	119.00
Cl1—C54—C55	119.86 (15)	C53—C52—H52	119.00
C53—C54—C55	121.2 (2)	C52—C53—H53	121.00
C54—C55—C56	119.07 (17)	C54—C53—H53	121.00
C51—C56—C55	121.26 (18)	C54—C55—H55	120.00
C1—C2—H2A	109.00	C56—C55—H55	120.00
C1—C2—H2B	109.00	C51—C56—H56	119.00
C3—C2—H2A	109.00	C55—C56—H56	119.00
			
C6—C1—C2—C3	−51.31 (19)	C4—C5—C51—C52	88.61 (17)
C11—C1—C2—C3	−174.65 (16)	C4—C5—C51—C56	−89.75 (19)
C12—C1—C2—C3	66.3 (2)	C6—C5—C51—C52	−147.05 (14)
C2—C1—C6—C5	58.91 (17)	C6—C5—C51—C56	34.6 (2)
C11—C1—C6—C5	−178.71 (13)	C5—C51—C52—C53	−178.71 (14)
C12—C1—C6—C5	−59.12 (17)	C56—C51—C52—C53	−0.3 (2)
C1—C2—C3—O3	−133.05 (17)	C5—C51—C56—C55	179.36 (15)
C1—C2—C3—C4	47.0 (2)	C52—C51—C56—C55	1.0 (2)
O3—C3—C4—C5	132.96 (17)	C51—C52—C53—C54	−1.1 (2)
C2—C3—C4—C5	−47.1 (2)	C52—C53—C54—Cl1	−177.24 (12)
C3—C4—C5—C6	51.79 (19)	C52—C53—C54—C55	1.8 (2)
C3—C4—C5—C51	178.83 (14)	Cl1—C54—C55—C56	177.93 (14)
C4—C5—C6—C1	−59.17 (18)	C53—C54—C55—C56	−1.1 (3)
C51—C5—C6—C1	176.20 (12)	C54—C55—C56—C51	−0.3 (3)

Symmetry codes: (i) *x*−1/2, *y*−1/2, *z*; (ii) −*x*, *y*, −*z*+1/2; (iii) *x*, *y*+1, *z*; (iv) −*x*, *y*+1, −*z*+1/2; (v) *x*+1/2, *y*+1/2, *z*; (vi) −*x*, −*y*+1, −*z*; (vii) −*x*, −*y*+2, −*z*; (viii) −*x*−1/2, −*y*+3/2, −*z*; (ix) *x*, *y*−1, *z*; (x) −*x*, *y*−1, −*z*+1/2.

### Hydrogen-bond geometry (Å, °)

**Table:**

*D*—H···*A*	*D*—H	H···*A*	*D*···*A*	*D*—H···*A*
C4—H4B···Cg^ii^	0.99	2.60	3.5333 (18)	157

Symmetry codes: (ii) −*x*, *y*, −*z*+1/2.
